# Engravings of the Nerves, Copied from the Works of Scarpa, &c.

**Published:** 1829-07-01

**Authors:** 


**ENGRAVINGS OF THE NERVES, COPIED FROM THE WORKS OF SCARPA, SOEMMERING, &c. By *E. Mitchell*, Engraver; with an explanatory Letter-press, by *Dr. Knox*, Lecturer on Anatomy. In 4to Numbers, price 1s. Edinburgh, 1829, Maclachlan and Stewart.**

We have examined with attention the various works which have, of late, appeared on the subject of *descriptive anatomy;* we have also examined the illustrative plates which have accompanied these publications; and we have, in consequence, arrived at the conclusion, that Dr. Knox and Mr. Mitchell have effected that which the student of anatomy has so long desired ;—we have now a work which every tyro in the science may study with advantage, and *every* practitioner derive improvement from.

Dr. Knox has done much as a teacher of anatomy, and still more as an author, to improve and enlarge that science to which he has devoted his undivided attention; he has enriched the medical literature of the country by his correct translation of the laborious CLOQUET—he has published many useful memoirs on Comparative and Pathological Anatomy, and he is now engaged in presenting to the profession a valuable series of engravings, illustrative of the origin and distribution of the nerves.

Although we do not think that anatomical drawings or engravings are, of themselves, calculated to convey to the student that portion of anatomical information which he requires, we are nevertheless of opinion that they may prove of much service to him, as agents in directing him to prosecute his investigations on the dead body.

Scarpa, Mascagni, and Cloquet, have given to the world representations of the different textures, structures, and parts of the human body, and they have done so with an accuracy that cannot be questioned—but their works are expensive, and are, therefore, beyond the reach of ordinary students.

Dr. KNOX, with proper attention to the interests of his pupils, has caused that eminent engraver, Mr. E. MITCHELL, to deliver, on a moderate, though sufficiently large scale, the most useful of the plates of these celebrated anatomists, and, what is of more consequence, he has not followed the example of other modern anatomists and engravers, in endeavouring to *alter and amend, without acknowledgment,* the productions of those to whom, in his title-page, he honestly confesses that he is indebted.

Appended to the engravings, Dr. K. has placed an excellent and minute description of the parts delineated. The first, second, and third plates are most accurate copies from the magnificent engravings by Anderloni, that embellish the valuable work of Scarpa; and we have no hesitation in affirming that they afford true and just views of the course and connections of the hypo-glossal—glosso-pharyngeal and cardiac nerves. The fourth plate, which is taken from Soëmmering, exhibits the base of the brain, with the origin of the cerebral nerves of their natural size.

We feel confident, from what has been stated, that this publication will find its way into the library of every student, and into the hands of all those medical practitioners who do not possess the original works.

As we are anxious that the merits of the undertaking should be fully appreciated, we shall notice the future Numbers as they appear, so that the profession may be enabled to judge of the extent of its bearings.

